# Phosphatase inhibitor, sodium stibogluconate, in combination with interferon (IFN) alpha 2b: phase I trials to identify pharmacodynamic and clinical effects

**DOI:** 10.18632/oncotarget.563

**Published:** 2011-12-22

**Authors:** Taolin Yi, Paul Elson, Masato Mitsuhashi, Barbara Jacobs, Emese Hollovary, G. Thomas Budd, Timothy Spiro, Pierre Triozzi, Ernest C. Borden

**Affiliations:** ^1^ Taussig Cancer Institute, The Cleveland Clinic, Cleveland, OH, USA; ^2^ Department of Immunology of Lerner Research Institute, The Cleveland Clinic, Cleveland, OH, USA; ^3^ Hitachi Chemical Research Center, Inc., Irvine, CA, USA

**Keywords:** Cancer, phase-I-trial, phosphatase-inhibitor, IFN-α2β, SSG

## Abstract

Since sodium stibogluconate (SSG) inhibited phosphatases including SHP-1 and augmented anti-tumor actions of IFN-α2b *in vitro* and in mice, two Phase I trials of SSG/IFN-α2b combination were undertaken to evaluate safety and target inhibition. Escalating doses of SSG (200-1200 mg/m^2^) and fixed doses of IFN-α2b (3x10^6^ units/m^2^) with or without chemotherapy (dacarbazine, vinblastine, cisplatin) were evaluated for side effects and impact on SHP-1 phospho-substrates and IFNα-stimulated-genes (ISGs) in peripheral blood in 40 patients with metastatic melanoma, soft tissue sarcomas, gastrointestinal stromal tumors, and breast or colorectal carcinomas who did not have other established treatment options. Common adverse events were bone marrow suppression, fatigue, gastrointestinal upset, and asymptomatic lipase elevation (n=13); the latter was dose related and mostly after 10d of SSG/IFN-α2b in combination. Levels of SHP-1 substrates (pSTAT1, pSTAT3, pLck and pSlp76) were increased (up to 3x) in peripheral blood cells following SSG with no potentiation by combination with IFN-α2b. Representative ISGs in peripheral blood were induced after IFN-α2b at 4 and 24 hrs with selective modulations by combination. The median time on trials was 2.3 months (10-281d) with no objective regression of disease. Alive at 1y were 17/40 (43%) patients and after 2y were 8/40 (20%) following treatment initiation. These data demonstrate that SSG impacted signal molecules consistent with PTP inhibition and was tolerated in combination with IFN-α2b. Phase II investigations of SSG could safely utilize doses of up to 1200 mg/m^2^ of SSG for up to 10d alone or in combination with IFN-α2b with or without chemotherapy.

## INTRODUCTION

PTPs are intracellular enzymes that act as the biochemical counterparts of tyrosine kinases [1]. Since their substrates are signal transducers activated or inactivated by phosphorylation to control cell growth, death or other functions, PTPs are key switches in signaling cascades that determine the fate of different cell types. In particular, the PTP SHP-1 [src-homology 2 phosphatase-1, PTP^1^C, HCP, SHPTP-1, PTPN^6^] has been implicated as an attractive drug-targeting candidate by studies from our laboratory and others [2-6]. Expressed predominantly in cells of hematopoietic lineages [2-6], SHP-1 has been established as a key negative regulator of cytokine signaling and immune cell activation through detailed studies of mouse models of genetic SHP-1 deficiencies [7-10]. Accordingly, targeting SHP-1 with inhibitors might augment the efficacy of cytokine therapy and immunotherapy, which are in clinical use for cancer treatment. In contrast to the significant numbers of protein kinase inhibitors approved by FDA or under-pre-clinical and clinical evaluation for cancer treatment, few PTPs inhibitors have demonstrated pre-clinical anti-tumor activity and entered clinical trial for cancer.

Our prior studies had identified sodium stibogluconate (SSG), a drug used for nearly 60 years to treat visceral leishmaniasis in humans [11], as a potent inhibitor of multi-PTPs that include SHP-1 and other PTPs critical in negative regulation cytokine signaling and immunity [12-14]. Targeting intracellular PTPs by SSG was suggested by the reduced PTP activity of SHP-1 and SHP-2 from cells cultured in the presence of SSG (10 mcg/ml) [13, 14]. At clinically achievable level of the drug when administered at half the currently recommended dose (10 mg/kg body weight), SSG inhibited recombinant SHP-1 (100%), SHP-2 (80%) and PTP1B (70%) [12, 15]. Selectivity was indicated by its limited impact on recombinant MKP1 PTP under comparable conditions [12]. It is worth noting that all cancer therapeutic kinase inhibitors target multi-kinases. This may provide corresponding multiple impacts against the redundant pro-cancer mechanisms *in vivo* and could be critical for clinical efficacy [16]. Multi-PTPs inhibitors may have clinical potential via a similar mode of operation and warrant investigation.

A negative regulatory role of SSG-sensitive PTPs in the signaling by IFNs was established in mutant mice in which the expression of the PTPs was abolished individually by genetic mutation or through gene-knockout [17-19]. Cells from these mutant mice had marked increases in response and tyrosine phosphorylation of IFN-alpha2 signaling molecules (e.g., Stat1) in comparison to control cells from normal mice [17-19]. Stat1 protein was activated through tyrosine phosphorylation following IFN-alpha2b stimulation to form ISGF3 (in complex with other molecules) which subsequently binds to ISRE to regulate gene expression[20]. Consistent with targeting the negative regulatory PTPs, SSG augmented IFN-alpha2b induced tyrosine phosphorylation of Stat1 in human lymphoma cell line [14]. Augmentation of IFN-alpha2b induced Stat1 tyrosine phosphorylation by SSG was also defined in WM9 human melanoma cells [14]. Enhanced IFN-alpha2b signaling brought about by SSG in cancer cells was coincident with the inhibition of specific intracellular target PTPs by SSG [14]. Anti-proliferative activity of IFN-alpha2b was potentiated by SSG in cancer cell lines of different tissue types [14]. Median effect analysis verified that SSG and IFN-alpha2b interacted in a synergistic manner (CI < 1)[21]. The ability of SSG to enhance significantly the anti-tumor effects of IFN-alpha2b *in vivo* was demonstrated in a mouse model with eradication of IFN-alpha2b-refractory tumors at a tolerable dose of the drug [14].

These studies, together with the safety of SSG in clinical use [15], provided the basis for Phase I trials of the combination of IFN-alpha2b and SSG, which as a multi-PTPs inhibitor has the potential to augment the anti-cancer action of the cytokine. Two Phase I trials were performed in similar patient populations. The objectives were to establish a safe dose of SSG to be used in conjunction with IFN-alpha2b for Phase II studies and to identify evidence of SHP-1 inhibition and any antitumor activity.

## RESULTS

### Patient Characteristics and Treatment Administration

Two trials with similar objectives were conducted. As described above, the first assessed SSG and IFN-alpha2b alone and then in combination and the second SSG and IFN-alpha2b, again alone and in combination, followed by cytotoxic chemotherapy. Since the patient populations and adverse events from IFN-alpha2b and SSG in the two trials were alike, except as noted below, results were summarized together. Entered in total were 40 patients with metastatic malignancies (melanoma n=29, soft tissue sarcomas n=5, gastrointestinal stromal tumors n=3, breast carcinoma n=1, and colorectal carcinoma n=2). These were patients for whom therapies of established effectiveness did not exist (prior radiation had been received by 13 and prior chemotherapy by 27). They were of median age of 53 (range 28-79), more of male gender (n=24), and mostly Caucasians (n=38). All patients were eligible and of ECOG performance status 0 or 1.

### Side effects

The most common instances of adverse events, worse than mild and considered possibly related to treatment from the combined continuous administration of the treatment regimens in the two trials, were granulocyte reduction (n=21), fatigue (n=22), gastrointestinal upset (n=14), fever (9), platelet reduction (n=12), anemia (n=11), lipase elevation (n=13), and hypokalemia (n=3) (Table 1). At least one attributed severe or life-threatening event occurred in 27 (68%) of patients, which were most frequently the expected bone marrow suppression from IFN-alpha2b ± chemotherapy (n=15 patients). Other severe or life-threatening adverse events associated with treatment, all ones previously associated with these drugs on other trials, were instance of lipase elevations (n=5), hypokalemia (n=3), and fatigue (n=3) (Table 1).

**Table 1:** Adverse Events Grade >2 by Study and Overall*

	2Y06 (SSG+IFN)			3Y06 (SSG+IFN+Chemo)			Both Studies combined		
		(n=18) grade			(n=22) grade			(n=40) grade	
Toxicity	2	3	4	2	3	4	2	3	4
**Granulocytopenia**	4	3	2	6	3	3	10	6	5
**Thrombo cytopenia**	5	1	-	3	3	-	8	4	-
**Anemia**	7	-	-	3	1	-	10	1	-
**Fatigue**	8	1	-	11	2	-	19	3	-
**Fever**	2	-	-	7	-	-	9	-	-
**G.I. upset**	2	-	-	9	3	-	11	3	-
**Elevated lipase**	2	5	2	2	1	1	4	6	3
**Hypokalemia**	-	-	-	-	3	-	-	3	-

* Attributed (at least possibly) to treatment. Of greatest severity by patient while on treatment

Asymptomatic lipase elevations (n=13) occurred most commonly after 10d of SSG in combination with IFN-alpha2b and were related to dose (*p*=0.04 by Fisher's exact test of <800 mg/m^2^ to > 800 mg/m^2^). Neutropenia and thrombocytopenia resulting from IFN-alpha2b at the highest dose of SSG (1200 mg/m^2^) when compared to the lowest doses (200 or 400 mg/m^2^) were not significantly potentiated by the addition of SSG (data not shown).

Progressive disease was the cause for terminating treatment in 28 patients. Five patients completed all planned cycles of treatment. Five patients were removed from study for adverse events (n=2 hypokalemia and one each of grade 4 lipase elevation, recurring neutropenia, and recurring nausea and vomiting). On a fourth cycle of treatment on the combination chemotherapy program after three relatively uncomplicated prior cycles, one patient died at a local hospital of a intra-cerebral event that could have been related to underlying disease or to treatment. Regardless of attribution 16/40 patients were assessed as having a one level decline in ECOG performance status and two patients as having > 2 level declines before removal from study. Median weight loss was 2.3 kg (mean loss of 3.0±3.5SD) kg (range -14.6 to +3.0 kg). Although progressive malignancy may have contributed, 13 patients lost more than 5% (grade 1) from starting weight during the period on treatment. Anorexia worse than mild was, however, reported in only two patients.

### Peripheral blood cellular phospho-proteins

**Figure 1:**
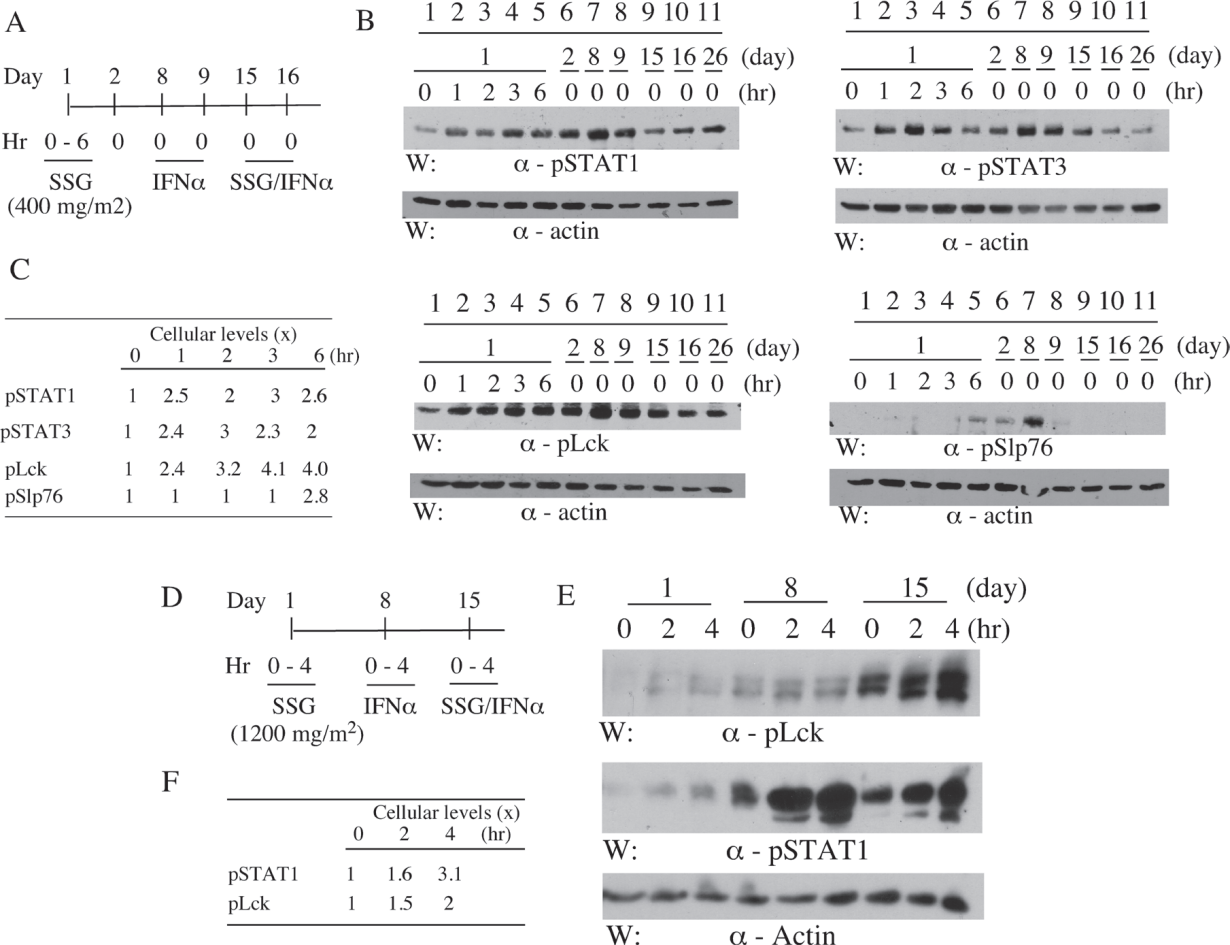
SSG modulates peripheral blood cell phospho-proteins in patients Peripheral blood samples were collected pre- and post-treatments from patients as indicated (A and D). Selective phospho-proteins in the samples were evaluated by SDS-PAGE/Western blotting with antibodies as indicated (B and E). The relative levels of the phospho-proteins were quantified by densitometry (C and F).

Given the putative targeting of PTPs by SSG, the impacts on selective phospho-proteins in peripheral blood cells were investigated in two cases (Fig 1). The selected molecules are reported substrates of the SSG-sensitive phosphatases and essential for T cell activation (pLck, pZap70 and pSlp76) or for IFN-alpha signaling (pSTAT1) [18, 22-26]. SSG monotherapy increased pSTAT1 levels (up to 3 x) (Fig 1C and F), treated at 400 mg/m^2^ and 1,200 mg/m^2^ respectively (Fig 1A and D). It was evident within 1 hr post-treatment with durations up to 6 hrs (Fig 1B). Similar increases in pSTAT3 and pLck levels following SSG were detected (Fig 1B) whereas increases of pSlp76 were evident at later time points (Fig 1B). A prolonged impact up to day 8 was apparent for pSTAT1, pLck and pSlp76 following a single dose of SSG on day 1 (Fig 1B, comparing lane 1 and 7). However, there was no obvious augmentation of IFN-alpha-induced pSTAT1 or pSTAT3 by SSG (Fig 1B and E) although pLck levels were markedly higher following SSG/IFN-alpha co-treatment (Fig 1E). The levels of pZap70 and pLAT were undetectable under the experimental conditions (data not shown).

### ISG product induction

**Figure 2:**
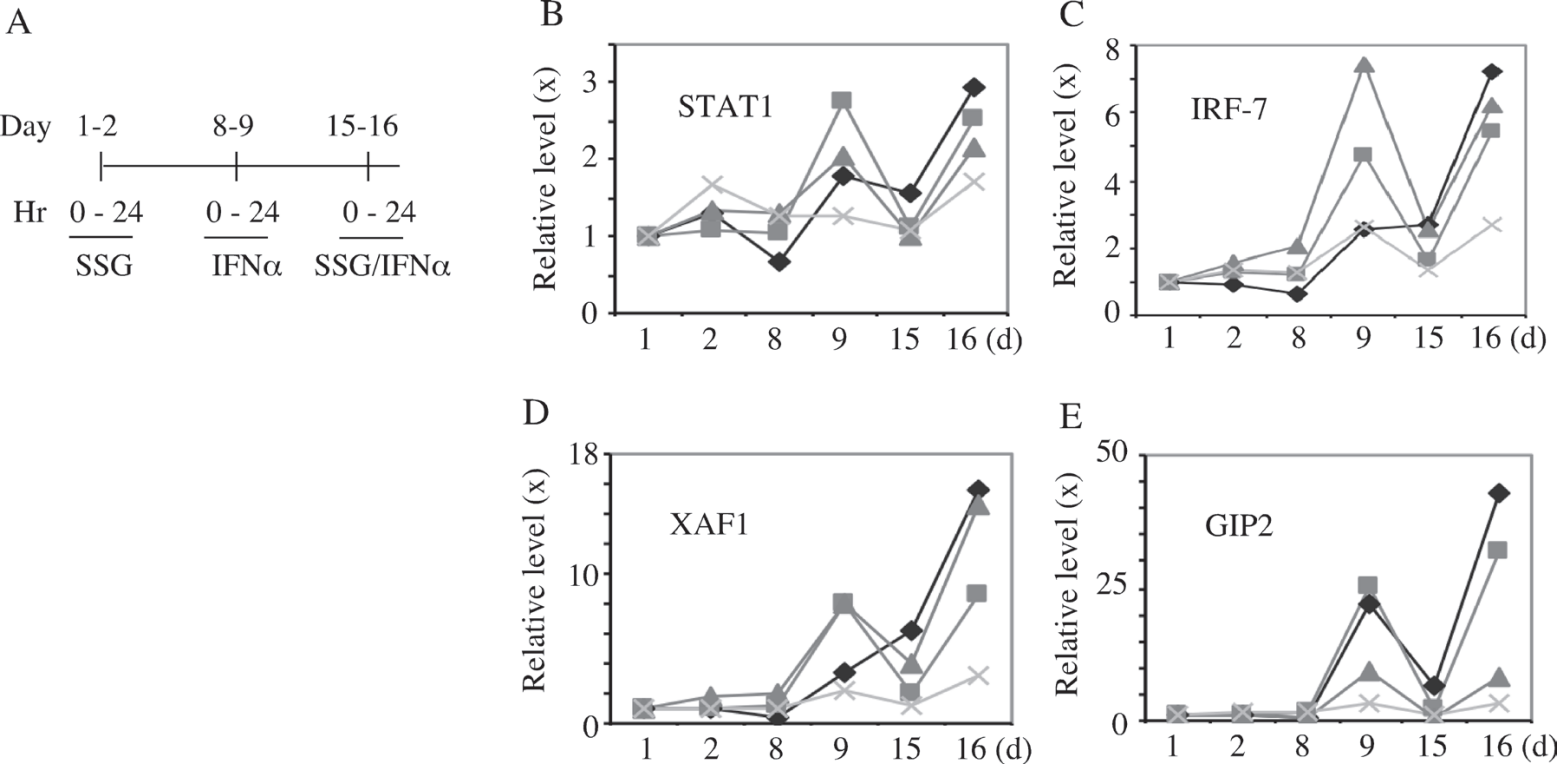
Peripheral transcripts levels of selective ISGs in patients Peripheral blood samples were collected pre- and post-treatments from 4 patients as indicated (A). The relative levels (fold change) of transcripts for STAT1, IRF-7, XAF1 and GIP2 were quantified by quantitative RT-PCR (B-E). Each symbol in panel B-E indicates a different patient.

To determine whether SSG augmented expression of RNA of ISGs, 4 patients were assessed for changes in STAT1, IRF7, XAF1, and G1P2 at 24 hrs after SSG at 800 or 1200 mg/m^2^, IFN-alpha2b, or the combination. These confirmed expected induction at 24 hours after IFN-alpha2b on day 9 but no consistent broad ISG increase after the combination (Fig 2). RNA samples from three other patients treated with SSG at 1200 mg/m^2^ were assessed after 4 hours each treatment identified increases in STAT1, IRF7, XAF1, G1P2, TRAIL, CXCL10, and AIM2 after IFN-alpha2 alone but with no differing results to suggest general broad potentiation of gene expression by SSG with IFN-alpha2 (data not shown). RNA expression levels of a panel of ~ 50 genes with immune modulatory potential, whose level might be affected by SSG or IFN-alpha2b were also assessed with no evidence of general potentiation (Supplemental Table 1).

**Figure 3:**
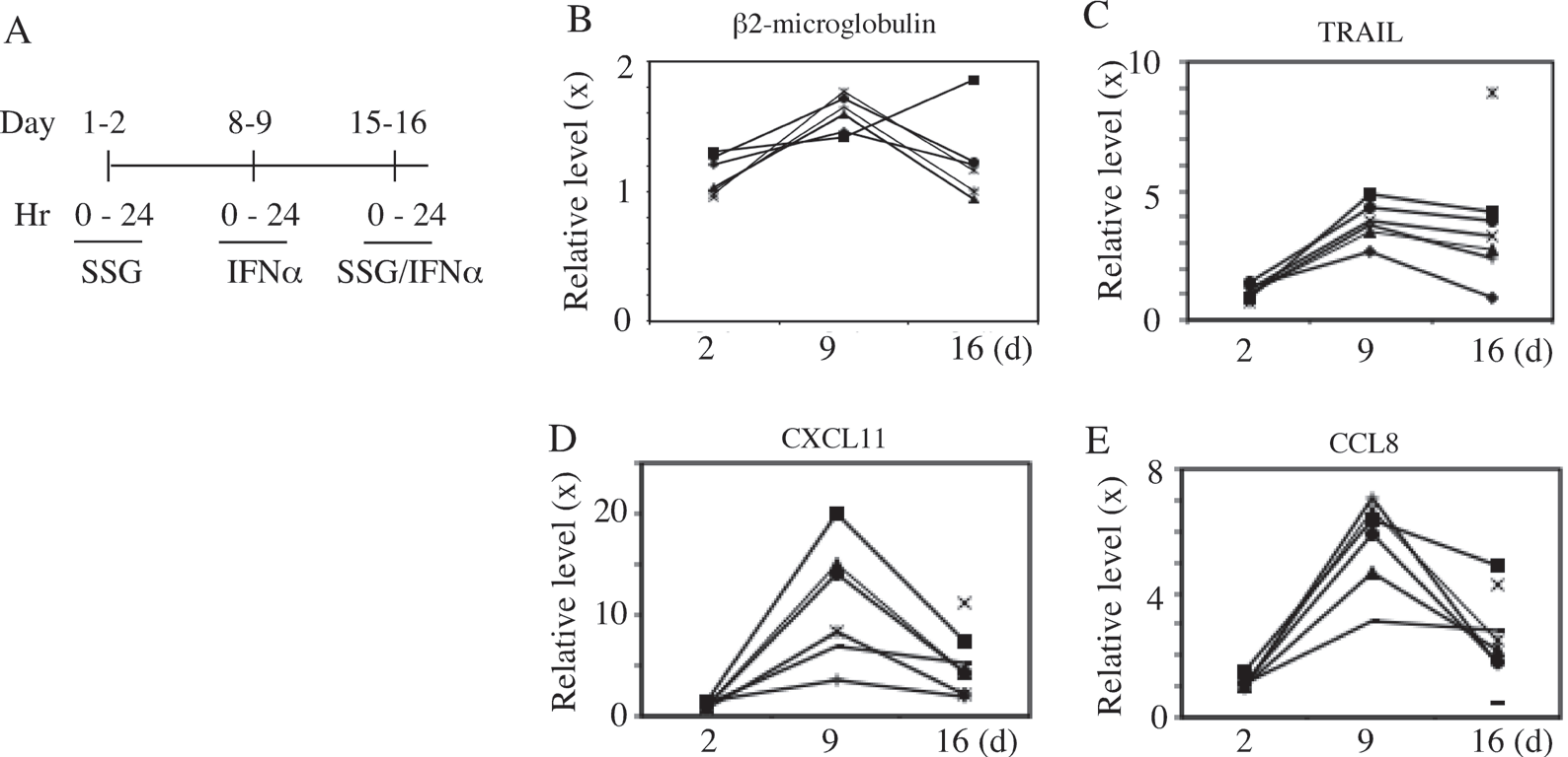
Peripheral protein levels of selective ISGs in patients Peripheral blood samples were collected at time points pre- and post-treatments from patients as indicated (A). The relative sera protein levels (ratio) of Beta2 microglobulin, TRAIL, CXCL11 and CCL8 were quantified by ELISA (B-E). Each symbol in panel B-E indicates a different patient.

To assess protein product change of ISGs, 7 patients treated at SSG 800 mg/m^2^ had representative ISG proteins, quantitated in serum by ELISA prior to treatment and 24 hours after each treatment. Beta2-microglobulin, TRAIL, CCL8, and CXCL11 all had expected increases 24 hours after IFN-alpha2 with return to near baseline after two days off and prior to SSG but no potentiation after the latter combined with IFN-alpha2b (Fig 3). Other proteins products of potentially modified gene expression assessed in serum after each treatment included IP-10 and IL-10; no consistent change was identified.

Antimony levels as a measure of achieved serum concentration of SSG, when measured 30 and 60 minutes after infusion at 800 mg/m^2^, exceeded 20 mcg/ml in 5/5 patients. We had identified this as a desired target concentration based upon complete inhibition of recombinant SHP-1 (100%)[12, 15].

### Treatment outcomes

No patient had objective regression of disease. The median time on trial was 2.3 months (range 10-281d). Although possibly influenced by prior treatment, patients receiving the chemotherapy regimen in addition to SSG and IFN-alpha2b were on study longer (median 81d) than those on only SSG and IFN-alpha2b (median 53d, *p* = 0.002 by Wilcoxon rank sum test). Of these patients with metastatic solid tumors, alive at 1y were 17/40 (43%) patients with 8/40 (20%) patients alive for >2 years after initiation of investigational treatment. The patients with > 2-year survival included six with melanoma, one with a gastrointestinal stromal tumor and one with hepatic angiosarcoma. All entered patients have now had treatment discontinued.

## DISCUSSION

SSG, a drug effective for chronic leishmaniasis [11], was a multi-PTPs inhibitor and augmented anti-cancer activity of IFN-alpha2b in mouse models [11-14]. Since it is selectively toxic to intracellular bur not free-living forms of the parasite [27-30] and intracellular survival of the pathogen in macrophages involves attenuation of cytokine signaling through PTPs [31], inhibition of PTPases may also provide an explanation for its antiparasitic activity. IFN-alpha2b-induced ISGF3 ISRE complex formation may be negatively regulated by PTPs. In support, SSG augmented IFN-alpha2b-induced ISGF3 ISRE complex formation in melanoma cells [14], consistent with an inhibitory action for PTPs. Additional evidence in support of the enhanced IFN-alpha2b signaling through the mechanism of PTP inhibition by SSG was derived from a cancer cell line that has defective intracellular IFN signaling and failed to respond to the combination of SSG and IFN-α2b[14, 32]. Following a 3-week course of IFN-α2b (5 x 10^5^ U/daily subcutaneously) alone, partial (~50%) growth inhibition of WM9 human melanoma tumors in nude mice resulted [14]. When combined with SSG (12 mg/day subcutaneously, IFN-alpha resulted in tumor regression and complete eradication of the tumors by the third week of study with long term tolerance for more than 10 weeks [14].

We sought to establish clinical safety of SSG alone and in combination with IFN-alpha2b and to identify evidence of SHP-1 inhibition. Forty patients with Stage IV metastatic malignancies were treated on the two protocols of Phase I design to identify toleration of doses of the investigational agent SSG escalated from 200-1200 mg/m^2^ with either a fixed dose IFN-alpha2b or in combination with fixed doses of IFN-alpha2b, dacarbazine, vincristine, and cisplatin. Of adverse events expected from previous investigations with SSG (hypokalemia, increased amylase, and increased lipase), only increased lipase and hypokalemia occurred in a substantial number of patients (grade 3 or 4 elevations in 22.5 and 7.5% of patients respectively). The asymptomatic, reversible lipase elevations were dose related (*p* = 0.04). Many of the grade 3 or 4 adverse events that did occur may have been due to progressive disease or the other drugs utilized rather than the investigational agent SSG. With appropriate assessment for adverse events, future investigations might consider utilizing doses of 1200 mg/m^2^ of SSG intravenously for up to 10d in combination with IFN-alpha2b with or without chemotherapy. Similar to our findings of acceptable tolerance of SSG in combination with IFN-alpha2b in cancer patients were findings in a concomitant study of similar design [33]. Both trials found that Phase II investigation of SSG could safely utilize doses > 800 and potentially 1200 mg/m^2^ for up to 10d in combination with IFN-alpha2b and, based upon our results, with addition of chemotherapy. Thus clinical use of a PTP inhibitor to potentiate other anti-tumor modalities is feasible.

IFN-alpha2 is established for treatment of hepatitis C virus, melanoma, and other malignancies [34]. IFNs have potent and pleiotropic gene regulatory effects in melanoma, antitumor activity for syngeneic murine melanomas, human melanoma xenografts, and effectiveness in patients with resected primaries at high risk for recurrence [34, 35]. Identifying and dissecting the relative role of the genes induced by IFNs that modulate pleiotropic effects of proteins on the tumor cell surface, alter levels of receptors for other cytokines, and activities of enzymes and cytokines that modulate cellular growth, function, and apoptosis, remains under investigation for targeting anti-tumor effects [36]. RNA expression of most ISGs did not seem to be potentiated by SSG but augmentation of selective ISGs may result as suggested by the heightened levels of XAF1 and GIP1 (Fig 2). This would need further confirmation and, if verified in larger studies, could be of specific ISGs for defining effects of SHP-1 inhibition.

SHP-1 is a critical negative regulator in anti-tumor immune cells that include T cells [37], NK cells [38], dendrocytes and macrophages [39]. Importantly, the PTP controls T cell activation [40] by reducing the sensitivity of the T cell receptor (TCR) to antigen [41, 42] and terminates TCR signals by dephosphorylating and inactivating essential components of TCR signaling cascade (e.g., Lck, Zap70 and Slp76) [43]. In NK cells, SHP-1 is coupled to inhibitory receptors for MHC class I antigen to prevent NK cell activation [44]. SSG was shown by our laboratory to inhibit SHP-1 [12], synergize with IFN-alpha to cure melanoma tumors in mice [14], and interact with IL-2 in anti-tumor action via a T cell-dependent mechanism [45]. An independent group demonstrated the capacity of SSG *in vitro* to help reversing T cell anergy among tumor infiltrating lymphocytes (TILs) from human melanoma, renal cancer and non-small cell lung carcinoma [46]. This is of particular interest given the reported suppression of TCR signaling and lytic function of CD8^+^ TIL cells by SHP-1 [47].

In this clinical trial, the target serum level of SSG for SHP-1 inhibition was exceeded by more than two folds. Despite what may have been limitations in assay methodologies for detecting labile phosphor-proteins, a suggestion that prolonged phosphoryation of T and NK cell signaling proteins inhibited by phosphatases may have resulted (Fig 1). In the parallel study patients receiving the higher doses (≥900 mg/m^2^) of SSG had a significantly lower number of Treg cells and myeloid dendritic cells together with a higher percentage of natural killer (NK) cells that synthesized perforin and of plasmacytoid dendritic cells (pDC) that secreted IFN-gamma in response to activation [33]. These findings suggest that in humans in addition to mice, SSG can have modulatory activity influencing innate and acquired immunity.

PTPs include enzymes that are oncogenic, tumor suppressive or immune regulatory [1, 3, 5, 48-50]. PTPs are thus key players in human malignancies and may have potential for developing inhibitors as novel cancer therapeutics [1, 5, 49-51]. Our data suggested tolerance of a SHP-1 inhibitor, SSG, in combination with IFN-alpha2b in cancer patients with evidence of immune regulatory activity in this study and one undertaken in parallel. Clinical inhibitors of SHP PTPs other than SSG have not been evaluated. These findings support developing more potent and selective PTP inhibitors for cancer treatment. Indeed, several small molecule inhibitors for selective PTPs, more potent than SSG, have been identified in recent studies and have had significant anti-tumor activities in pre-clinical models [52, 53]. Further development and evaluations of other PTPs inhibitors are warranted and have promise as targeted therapeutics for effective and safe cancer treatments.

## PATIENTS AND METHODS

### Inclusion and exclusion criteria

All patients met the following eligibility criteria: histological diagnosis of metastatic malignancy, measurable disease as defined by the NCI Response Evaluation Criteria in Solid Tumors (RECIST) guidelines, performance status (ECOG) of 0-2, recovered > 3 weeks from the any radiation therapy, not have received any adjuvant or metastatic disease IFN-alpha2 ≤ 4 months prior, no other established treatment options, and no major surgery within 28 days, together with granulocyte count ≥ 1.5 x 10^9^/L, platelets ≥ 100 x 10^9^/L, creatinine <1.0 x upper limit normal (ULN), bilirubin<1.5 x ULN, AST <1.5 x ULN, and ALT <1.5 x ULN, and must have provided written informed consent as to the investigative nature of treatment in accordance with institutional and federal guidelines. Excluded were patients with uncontrolled CNS metastases in the prior 3 months, chronic infections, history of arrhythmia with baseline ECG abnormalities suggestive of conduction delay, i.e. 1^0^ or greater atrio-ventricular block complete or incomplete (QRS > 120 ms) bundle branch block, or suggestive of repolarization abnormalities, i.e. QTc > 0.48 sec, congestive heart failure, pregnant or lactating women, fertile women or men unless surgically sterile or using effective contraception, evidence of HIV or HBsAg, organ allografts, high dose glucocorticoids, age < 18, or history of severe psychiatric disorders.

### Treatment plan

Cohorts of patients were enrolled at dose levels of SSG of 200, 400, 800, and 1200 mg/m^2^/day on days 1-5 and 8-12 and IFN-alpha2b alone at a dose of 3 MU/m^2^ subcutaneously daily for 14d. The starting dose of SSG was chosen to be less than that used for daily treatment of visceral leishmaniasis (800 mg/m^2^/day) and the IFN-alpha2b dose to be substantially less than the maximally tolerated. To enable pharmacodynamic assessments of each agent alone, schedule and dose initiation was with a single administration of SSG on the first day, IFN-alpha2b beginning a week later for 5d, and the combination beginning on day 15 for two weeks. SSG dose was escalated with successive cohorts but not escalated within cohorts. Treatment for two weeks continued at 28d intervals until disease progression or dose limiting toxicity (DLT) occurred.

Both trials used a standard 3+3 dose escalation design to determine the maximal tolerated dose (MTD) of SSG, with escalation being based on the number of dose-limiting toxicities observed (grade >3 treatment-related adverse event by CTCAE 3.0 criteria that persisted despite medical treatment/prophylaxis, or grade 2 cardiovascular arrhythmia in the absence of hypokalemia or hypomagnesemia). That is, cohorts of three patients were initially treated at a particular SSG dose. If none of these patients experienced DLT, SSG was to be escalated to the next higher dose level. If two or more patients experienced DLT, the trial was to be stopped and the next lower dose defined as the MTD. If one patient had DLT, three additional patients were to be treated at that dose, with escalation occurring if no additional DLT was observed. Patients were assessed for adverse events weekly for 4 weeks, biweekly for 2 weeks, and then monthly. Measureable disease was assessed at each visit and by imaging every 2 months. Adverse events were measured and graded according to Common Terminology Criteria for Adverse Events (CTCAE) (NIH 2003).

After validation of lack of unexpected adverse events in the initial patients, a second protocol in combination with chemotherapy was initiated with parallel eligibility criteria (eligibility for Stage IV patients without measureable disease for up to four cycles of treatment added). This utilized dose levels of SSG of 200, 400, 800, and 1200 mg/m^2^/day for four days with IFN-alpha2b at a dose of 3 MU/m^2^ subcutaneously daily for 4d. Again for pharmacodynamic assessments, SSG and IFN-alpha2b alone and then in combination were initiated as a single administrations of SSG on the first day, beginning a week later 3 MU/m^2^ for 5d, and a chemotherapy combination initiated beginning a week thereafter for 2d with cisplatin at a dose of 30mg/M^2^ IV, vinblastine 2.0mg/ M^2^ IV, and dacarbazine 350mg/m^2^ IV with each chemotherapy dose given daily. Cycles were repeated every 28d.

The studies were conducted under IND # 68881 from the FDA with the clinical grade SSG for investigational study in the US provided by Albert David Inc, Calcutta and with commercially available IFN-alpha2b from Schering Merck. The protocols (IRB 2Y06 and IRB3Y06) were conducted with approval from and under the auspices of the Investigational Review Board of the Case Comprehensive Cancer Center and were registered as CT.gov NCT00311558 and NCT00498979 respectively.

### ELISA assays for ISG products

Beta_2_-microglobulin (R&D Systems, Minneapolis, MN) was quantitated in patients' sera using a competitive binding enzyme immunoassay. TRAIL, CXCL11, and CCL8 (MCP-2) (RayBiotech, Raitan, NJ) were quantitated in frozen stored patients' sera using individual quantitative sandwich enzyme immunoassays for batched samples. The lower limits of sensitivity for the ELISA assays ranged from 0.2 ug/ml for Beta2 microglobulin, 2.9 pg/ml for TRAIL, 14 pg/ml for CXCL11 and 1.5 pg/ml for CCL8.

### RNA collection and analyses

Blood was collected in PAX tubes (PreAnalytiX Inc., Franklin Lakes, NJ) at pre treatment and day 8, and RNA was prepared using the PreAnalytiX Blood RNA kit according to manufacturer's instructions. cDNA was prepared using the SuperScript III First-Strand Synthesis System (Invitrogen Inc. Carlsbad, CA) according to instructions of the manufacturer. Selected gene expression was assessed by iTaq SYBR (BioRad and ABI7900, cycler [54]. Primer sequences were G1P2: CAAATGCGAC GAACCTCTGA, CCGCTCACTT GCTGCTTCA, XAF1: CCTAGAGGAG ATAAAGCAGC CTATGA, AAGCTAACCA CCGGCATTTCT, IRF7: TCCCCACGCT ATACCATCTA CCT, ACAGCCAGGG TTCCAGCTT and STAT1: GTGGAAAGAC AGCCCTGCAT, ACTGGACCCC TGTCTTCAAG AC. GAPH was used to normalize CT values and fold expression was calculated based on pretreatment CT values using the ddCT formula.

### Antimony quantification

Antimony, a constituent of SSG [55], has been used to assess SSG serum concentration. It was quantitated by ARUP at the University of Utah by inductively coupled plasma/mass spectrometry, reference range 0-6 microg/L.

### Phospho-proteins in peripheral blood

Heparinized peripheral blood samples were obtained by vein-puncture per clinical trial protocols approved by the Institutional Review Board (IRB) of Cleveland Clinic. For evaluation of phospho-proteins, the samples were diluted (5x) with cold hypotonic solution (10 mM Tris, pH 7.4; 10 mM NaCl; 0.2 mM Na_3_VO_4_) to lyse RBC and washed 2 x with the solution. The WBC pellets were lysed in cold lysis buffer (1% NP40, 50 mM Tris, pH 7.4, 150 mM NaCl, 20 mM NaF, 0.2 mM Na_3_VO_4_ and 1 mM Na_3_MO_4_) containing a cocktail of proteinase inhibitors (Sigma, 1 tablet/10 ml). The lysates were cleared by centrifuging (14,000 rpm, 10 min) in a microfuge at 4^0^C to remove insoluble parts, mixed with equal volume of 2 x SDS-PAGE sample buffer, boiled for 5 min and analyzed by SDS-PAGE/Western blotting. Relative intensities of phosphotyrosine bands were quantified through densitometry analysis. Antibodies against pStat1 (New England Biolab), pStat3 (Santa Cruz Biotech), pLck (Cell Signaling), pZap70 (BD Biosciences), pSlp76 (BD Biosciences) and pLat (BD Biosciences) were from commercial sources

## Supplementary Tables

Supplementary Table 1
