# Supplementary material for: Phosphatase inhibitor, sodium stibogluconate, in combination with interferon (IFN) alpha 2b: phase I trials to identify pharmacodynamic and clinical effects
Source: Oncotarget. 2011 Dec 22;2(12):1155–64. doi: 10.18632/oncotarget.563 (PMC3282074; doi:10.18632/oncotarget.563)
Supplement: Supplementary Table 1 [file oncotarget-02-1155-s001.docx]

Supplemental Table 1.

| RNA products of genes assessed without evidence of change from baseline by SSG, IFN-alpha2b, or the combination | | | |  |  |
| --- | --- | --- | --- | --- | --- |
|  |  |  |  |  |  |
|  | **Genes 4 hours after drug** | |  | **Genes 24 hours** | |
|  | T2 | Granulysin | | CXCL9 |  |
|  | IFNG | JAK1 |  | ZAP70 |  |
|  | iNOS-1 | ICOS |  | ICOS |  |
|  | Perforin | ZAP70 |  | TNFSF2 |  |
|  | IL-2 | IKB |  | BST2 |  |
|  | IL-12B | TCR |  | CXCL10 |  |
|  | GMCSF | PDCD |  | SOCS1 |  |
|  | T6 | DUSP |  | IFNG |  |
|  | SOCS1 | GBP |  | AIM2 |  |
|  | STAT3 | IFI6 |  | IL-2 |  |
|  | LCP | Granzyme B | | Granulysin | |
|  | IL-23A | CXCL10 |  | GBP |  |
|  | SHP1 | AIM2 |  | FOXP3 |  |
|  | Lck | IL-12A |  | Perforin |  |
|  | FOXP3 | BST2 |  | Granzyme B | |
|  | CD8A | T5 |  | GMCSF |  |
|  | T10 | X9 |  | IL-12 |  |
|  | IFI27 | X2 |  |  |  |
